# Temperature sensitive point mutations in fission yeast tropomyosin have long range effects on the stability and function of the actin-tropomyosin copolymer

**DOI:** 10.1016/j.bbrc.2017.10.109

**Published:** 2018-11-25

**Authors:** Chloe A. Johnson, Holly R. Brooker, Irene Gyamfi, Jennifer O'Brien, Brogan Ashley, Jodie E. Brazier, Annette Dean, James Embling, Elisabeth Grimsey, Alice C. Tomlinson, Elliot G. Wilson, Michael A. Geeves, Daniel P. Mulvihill

**Affiliations:** School of Biosciences, University of Kent, Canterbury, Kent, CT2 7NJ, UK

**Keywords:** Acetylation, *Schizosaccharomyces pombe*, Fission yeast, Actin cytoskeleton, Cdc8, Thermal stability

## Abstract

The actin cytoskeleton is modulated by regulatory actin-binding proteins which fine-tune the dynamic properties of the actin polymer to regulate function. One such actin-binding protein is tropomyosin (Tpm), a highly-conserved alpha-helical dimer which stabilises actin and regulates interactions with other proteins. Temperature sensitive mutants of Tpm are invaluable tools in the study of actin filament dependent processes, critical to the viability of a cell. Here we investigated the molecular basis of the temperature sensitivity of fission yeast Tpm mutants which fail to undergo cytokinesis at the restrictive temperatures. Comparison of Contractile Actomyosin Ring (CAR) constriction as well as cell shape and size revealed the *cdc8.110* or *cdc8.27* mutant alleles displayed significant differences in their temperature sensitivity and impact upon actin dependent functions during the cell cycle. *In vitro* analysis revealed the mutant proteins displayed a different reduction in thermostability, and unexpectedly yield two discrete unfolding domains when acetylated on their amino-termini. Our findings demonstrate how subtle changes in structure (point mutations or acetylation) alter the stability not simply of discrete regions of this conserved cytoskeletal protein but of the whole molecule. This differentially impacts the stability and cellular organisation of this essential cytoskeletal protein.

## Highlights

• Cloning, expression and characterisation of fission yeast temperature sensitive tropomyosin mutants.
• Detailed *in vitro* analysis on the impact of temperature upon these mutants.
• Comparison with in vivo impact of mutations upon actin ring function within the fission yeast.
• Demonstrates that subtle changes in structure alter the long range stability of Tropomyosin containing polymers.

## 1 Introduction

The ability to transiently perturb molecular interactions is invaluable in elucidating protein regulation and function in both a cellular and *in vitro* context. The yeasts provide excellent genetically tractable systems for exploring the cellular function of gene products. Their genomes can be rapidly modified to introduce labels or mutations of proteins using either directed or random genomic replacement approaches. These have been used to great effect and provided a unique insight into our understanding the molecular basis of cell cycle and cytoskeletal regulation [1], [2]. Through the generation of conditional mutants, protein function can be specifically repressed within a culture of growing cells. In the case of temperature sensitive mutations normal function continues while cells are cultured at a lower permissive temperature (e.g. 25 °C), but shifting the culture to a higher temperature (e.g. 36 °C), specifically perturbs the functionality of the mutant protein and hence disrupts its individual role in cell proliferation. These mutants are invaluable in the study of dynamic processes critical to the viability of a cell. However, the molecular mechanisms that underlie the temperature sensitivity have yet to be defined for the majority of these mutants, despite being isolated more than 40 years ago.

Tropomyosin (Tpm) is a highly conserved alpha-helical, coiled-coil protein that forms two parallel polymers along the surface of the actin filament, stabilising the actin filament and regulating its interactions with other cytoskeleton components [3], [4]. The Tpm helical coiled-coil structure is defined by a heptad repeat of residues within the polypeptide sequence. This promotes regular hydrophobic interactions along the coiled-coil to stabilise the dimer, and allows salt bridges to form between dimerising proteins. The propensity of Tpm to form end-to-end contacts and polymerise is significantly enhanced by N-terminal (Nt) acetylation, which changes the local charge, and stabilises the coiled-coil structure at the amino-terminus of Tpm [5], [6], [7].

Yeasts provide attractive model systems for studying diverse cellular processes. They contain the simplest mechanistic and regulatory systems to underpin cellular processes, which are often significantly more complex in metazoans. This simplification of cellular mechanisms is reflected in the yeast Tpm based regulatory system. Budding and fission yeasts each contain two functionally distinct Tpm isoforms compared to more than 40 in mammalian cells. *Saccharomyces cerevisiae* expresses two Tpm proteins, Tpm1 and Tpm2 [8], existing exclusively in Nt-acetylated forms in the cell, and each facilitate discrete cellular processes [9], [10]. In contrast, the fission yeast, *Schizosaccharomyces pombe*, contains a single essential Tpm, Cdc8 (Tpm^Cdc8^), which exists in both Nt-acetylated and non-acetylated forms within the cell, each of which associates with discrete sets of actin polymers to modulate their cellular function [11], [12]. Cells lacking functional Tpm^Cdc8^ fail to form a functional CAR and therefore fail to undergo cytokinesis. In addition they fail to deliver myosin V dependent cargoes to the normal sites of cell growth, and therefore lose polarity [13], [14].

In this study we investigate the molecular basis of temperature sensitivity of two fission yeast temperature sensitive tropomyosin mutants, *cdc8.27* and *cdc8.110*, to provide an insight into the structure-function relationship of this conserved cytoskeletal protein. *In vivo* analysis revealed each mutant disrupts the ability of the fission yeast cell to grow and divide in discrete ways. Differences in functionality were seen to correlate with positions of amino-acid substitutions and thermal stability of the mutant proteins. These data show that subtle changes in the stability of specific regions of this conserved protein differentially impact the stability and function of this essential cytoskeletal component.

## 2 Materials and methods

*Cell culture*: The yeast strains used in the study were h^−^ *cdc8*^+^; h^−^ *rlc1.gfp:kanMX6*; h^−^ *cdc8.110 rlc1.gfp:kanMX6*; h^−^ *cdc8.27 rlc1.gfp:kanMX6*. Cell culture and maintenance were carried out according to [15] using Yeast Extract supplement with amino acids (YES). All cells were maintained as early to mid-log phase cultures for 48 h (grown at 25 °C) before analyses were performed.

*Microscopy*: Imaging was undertaken as described previously [11]. Timelapse images of >20 cells of each strains undergoing cytokinesis were used to characterise AR formation and constriction at each temperature. Cell dimensions were calculated from images of >300 cells of each strain using a Matlab (Mathworks, Natick, USA) based automated image analysis programme, generated within this laboratory [16] with additional algorithms for measuring cell curvature (O'Brien et al., In preparation).

*Molecular Biology*: *cdc8* alleles were amplified from genomic DNA preparations of *cdc8.27* and *cdc8.110* cells, cloned into pGEM-T-Easy vector (Promega, Madison, USA) and sequenced. This procedure was repeated thrice from independent genomic preparations. pJC20*cdc8*^+^ was described previously [11]*. cdc8.A18T*, *cdc8.E31K, cdc8.A18T E31K* and *cdc8.129K* were synthesised as *Nde1* – *BamH1* fragments (ThermoFisher, Waltham, USA) and cloned into the pJC20 [17] bacterial expression vector. All constructs were sequenced prior to being used for recombinant protein expression.

*Protein Purification*: Tpm^Cdc8^ proteins were expressed from pJC20 based plasmids in either BL21 DE3 or BL21 DE3 pNatB [15] cells to produce protein in non-acetylated and Nt-acetylated forms respectively. Mid-log phase cultures were grown for 3 h with 100 mg/l IPTG. Cells were harvested, resuspended in 30 ml lysis buffer (20 mM Tris pH 7.5, 100 mM NaCl, 2 mM EGTA and 5 mM MgCl_2_), lysed by sonication and heated to either 85 °C (wild type) or 65 °C (mutant proteins) for 10 min. Debris and insoluble components were removed by centrifugation and nucleotides were removed from the resulting supernatant was incubated with 10 mg/l Bovine Pancreas purified DNase I (Sigma #69182) and RNase A (Sigma #R6513) at 4 °C for 1 h. After buffer exchange into FPLC loading buffer (5 mM Tris pH 7.0, 100 mM NaCl) the Tpm^Cdc8^ was subjected to 3 rounds of FPLC purification using 2 × 5 ml Pharmacia HiTrap-Q columns in tandem, by elution with a 0.1- 0.9 M NaCl gradient. After the final FPLC run the protein was resuspended in 5 mM Tris pH 7.0 and subjected to electrospray mass spectroscopy (Fig. S2), SDS-PAGE, and spectrophotometric analyses to determine mass, homogeneity, purity and protein concentration of each protein preparation (Tpm^Cdc8^ extinction coefficient at 280 nm - 2980 M^−1^ cm^−1^). Rabbit actin was purified as described previously [18].

*Circular dichroism* (CD): Measurements were made in 1 mm quartz cuvettes using a Jasco 715 spectropolarimeter. Tpm^Cdc8^ proteins were diluted in CD buffer (10 mM potassium phosphate, 5 mM MgCl_2_ pH 7.0) to a concentration of 0.4 mg/ml. CD buffer was supplemented with 500 mM NaCl unless stated otherwise in the text to minimize end to end polymerization of Tpm^Cdc8^. Thermal unfolding data were obtained by monitoring the CD signal at 222 nm with a heating rate of 1 °C.min^−1^. At completion of the melting-curve the sample was cooled at a rate of 20 °C.min^−1^. CD spectra are presented as differential absorption (ΔA). Melting curves are reported as fraction of unfolded protein by normalizing the CD signal between 10 and 50 °C.

*Actin binding assay*: Co-sedimentation assays were performed at 25 °C by mixing 10 μM actin with increasing concentrations of Tpm and then assaying the proteins present in the pellet and supernatant using SDS-PAGE as described previously [19], [20]. Briefly the gels are scanned to yield the relative density of the actin (loading control) and Tpm bands. The ratio of the densities of the Tpm band to the actin band provided an estimate of the fractional saturation of the actin by Tpm. Analysis of the binding isotherms using the Hill equation yields an estimate of free Tpm concentration required for half saturation of actin affinity (K_50%_). Values presented are an average of 3 indpendent experiments. This is not a true affinity measurement since Tpm is believed to polymerise on actin.

## 3 Results

The biophysical basis of the temperature sensitivity of two discrete alleles of the fission yeast tropomyosin, Tpm^Cdc8^, *cdc8.27* and *cdc8.110* [1] were examined. Sequence analysis revealed these alleles encode for proteins with amino acid substitutions at opposite ends of the Tpm protein. The *cdc8.27* allele contains a single point mutation, resulting in an E129K amino acid substitution towards the carboxyl terminus of the resultant protein, as reported [21]. In contrast sequence analysis revealed the *cdc8.110* allele encodes for a protein with 2 discrete amino acid substitutions, A18T and E31K, both within the amino region of the protein.

**Fig. 1:**
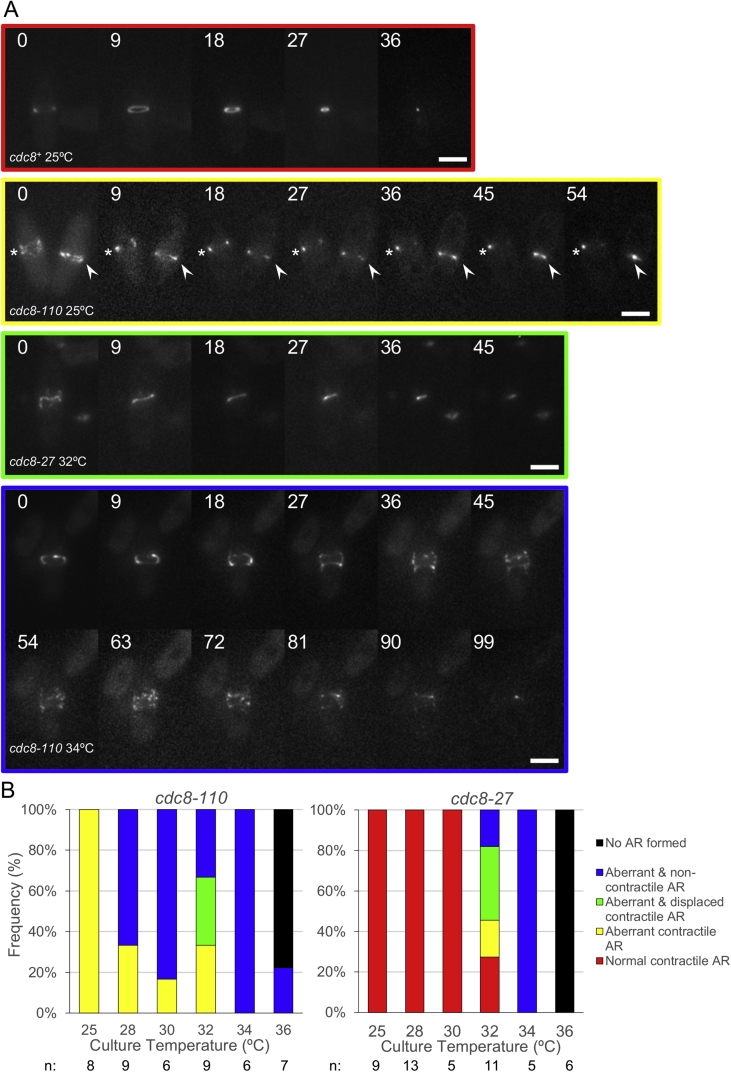
**CAR formation and constriction in cells containing temperature sensitive *Tpm***^***cdc8***^**alleles.** Mid log phase cells were mounted onto the microscope as described previously and held at the designated temperature for 20 min before undertaking observations. (A) Maximum projection of 21-z slice images from timelapse experiments (9 min between frames) where CAR formation and constriction was monitored in *rlc1.gfp* cells containing either a *cdc8*^+^, *cdc8.110,* or *cdc8.27* allele. 0 time denotes the start of the experiment and does not relate to other mitotic events. Cells formed either normal contractile rings (red border); aberrant rings that after a delay went on to constrict (yellow border - asterisk); aberrant rings that detached from the cortex and subsequently constricted to form a misplaced septum (green border); or aberrant rings that collapsed upon constriction (blue border). 9 min between frames. Scale bars–5 μm. (B) Histograms from analysis of CAR constriction of >45 cells from each cell type showing frequency of each Actin Ring (AR) phenotype described in (A) in *cdc8.110,* or *cdc8.27* cells when incubated at different temperatures. Colours are consistent with (A). n denotes number of cells examined at that temperature. (For interpretation of the references to colour in this figure legend, the reader is referred to the web version of this article.)

CAR formation and constriction was examined at a range of temperatures (from 25 °C and 36 °C) in *cdc8*^+^*, cdc8.27* and *cdc8.110* cells expressing fluorescent labelled myosin II regulatory light chain, *rlc1.gfp* [22]. This marker allele was chosen as, unlike the majority of other FP actin filament markers described to date, it has been reported to have no impact upon CAR formation or function [22], [23]. In contrast to wild type *cdc8*^+^ cells, which formed normal functional contractile CARs at each temperature examined (n > 80), cells containing the *cdc8.110* and *cdc8.27* demonstrated a variety of defects in the organisation and function of the contractile machinery (Fig. 1), which increased in severity as the temperature was increased.

*cdc8.110* mutant cells displayed a number of actin organisation defects, even at 25 °C, with CAR formation taking longer and being less organised in all cells examined at this permissive temperature (Fig. 1 - yellow outlined montage and bars). Aberrant CARs were observed to either slide along the cell cortex with its subsequent constriction resulting in a misplaced or misaligned septum (Fig. 1 -green outlined montage and bars), or disintegrate upon constriction (Fig. 1 - blue outlined montage and bars). The frequency of each CAR defect varied according to temperature in an allele specific manner. In contrast to *cdc8.110* cells which demonstrated a gradual increase in the frequency of defective CAR, *cdc8.27* cells grew normally and demonstrated normal CAR formation and function at temperatures up to 30 °C. Above this temperature, the majority of cells formed CARs that collapsed upon constriction. At 36 °C both mutant strains failed to form any discernible CAR structure and went on to form multinucleate dumb-bell shaped cells [1], [24]. In contrast polarity defects were only observed in *cdc8.110* mutant cells at lower permissive temperatures of 25 °C and 28 °C. Automated image analysis [16] revealed that at these temperatures *cdc8.110* cells were ∼10% shorter and 10% wider than both wild type and *cdc8.27* cells (*t*-test significance at >99% confidence), with a significant proportion (39%) displaying a bent cell morphology (Fig. S1). Both of these phenotypes are consistent with tropomyosin stabilised actin polymers having a direct impact upon either microtubule organisation and/or the regulation of polarised cell growth [25].

**Fig. 2:**
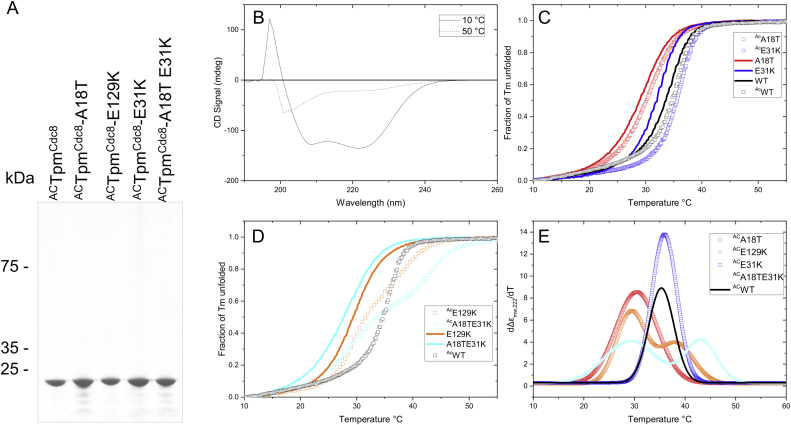
**CD spectra and melting curves.** (A) Coomassie-Blue stained SDS-PAGE gel showing purified acetylated wild-type and mutant Tpm^cdc8^ proteins. (B) CD spectra for unacetylated Tpm^Cdc8^ at 10 and 50 °C. Normalised CD signal at 222 nm for ^Ac^Tpm and unacetylated Tpm as a function of temperature (C) WT-, A18T- and E31K-Tpm and (D) WT-, E129K- and A18TE31K-Tpm. (E) First derivative plots for the four ^Ac^Tpms melting curves.

To determine how differences in the cooperative temperature effects related to the physical properties of the mutant Tpm^Cdc8^ proteins, bacterial expression constructs were generated. These contained cDNA of the mutant *cdc8.27* and *cdc8.110* alleles (Tpm^Cdc8.^E129K and Tpm^Cdc8.^A18T-E31K respectively) as well as *Tpm*^*cdc8*^ containing individual mutations from the *cdc8.110* allele (Tpm^Cdc8.^A18T and Tpm^Cdc8.^E31K). Wild type and mutant Tpm^Cdc8^ proteins were expressed and isolated from bacteria in both unmodified and Nt-acetylated forms [26]. The purity of each protein was confirmed by SDS-PAGE (Fig. 2A) and electrospray mass spectroscopy confirmed that the relative molecular mass of each protein was not only homogeneous but was also within 1 kDa of that predicted (Fig. S2).

In order to explore the impact each substitution has upon structure and thermal stability, each Tpm^Cdc8^ protein was subjected to CD analysis. Tpm folds into α-helices along its entire length, which is consistent with the broad negative peaks at 208 and 222 nm and a positive peak at < 200 nm observed for both wild type and mutant Tpm^Cdc8^ proteins (Fig. 2B). Spectra were collected between 190 and 260 nm before and after heating the proteins to 50 °C. Spectra obtained at 50 °C showed almost a complete removal of the positive peak at <200 nm, and a marked reduction in the signal intensity of the 2 broad negative peaks associated with all Tpm proteins, consistent with a shift from a folded to an unfolded state (Fig. 2B). The proteins were subsequently cooled to 10 °C and the CD spectra indicated a complete reversal of the unfolding as previously reported for Tpm^Cdc8^ [27]. The samples were then subjected to a further 2 melts to examine reproducibility. The spectra were equivalent for all Tpm^Cdc8^ proteins used, illustrating the full reversibility of unfolding.

Melting profiles measured at 222 nm were normalised and are shown in Fig. 2C and D. The data is presented as an average of the three unfolding profiles, as minimal variation was seen between the 3 melts for each Tpm protein. A first derivative plot was calculated from these data, to which a one or two Gaussian function was fitted, from which mid-points (T_m_) of melting were calculated (Fig. 2E, Table 1). The T_m_ of wild type Ac- and unAc-Tpm^Cdc8^ corresponds with previously published work [27]. In the absence of acetylation each mutant displayed a decrease in thermal stability. The A18T-E31K double mutation had the most dramatic effect, resulting in a decrease in T_m_ from 34.1 °C to 27.9 °C. The A18T and E129K mutants had similar T_m_s of 29.3 °C and 29.4 °C, respectively while E31K had the smallest effect on thermal stability, with a T_m_ of 32.2 °C. While amino-terminal acetylation of the wt and mutant Tpm^Cdc8^ resulted in an increase in thermal stability compared to the unacetylated proteins, it did so to varying degrees for each protein. Wild Type Tpm^Cdc8^ was stabilised by 1.3 °C. Acetylating the E31K mutant stabilised the protein by 3.7 °C resulting in a similar T_m_ to that of acetylated WT Tpm^Cdc8^ (35.9 °C and 35.4 °C, respectively). A18T was stabilised by a more modest 1.2 °C on acetylation.

**Table 1:** Summary of thermal properties of Tpm^Cdc8^ proteins.

	T_m_(°C) Tpm 0.5 M KCL	T_m_(°C) Ace-Tpm 0.5 M KCL	T_m_(°C) 0.1 M KCL	ΔH kJ·mol^−1^	ΔH Ace-Tpm kJ·mol^−1^
Tpm^Cdc8^	34.1	35.4	30.3/33.9	−455.8	−555.7
Tpm^Cdc8^-A18T	29.3	30.5		−315.4	−342.3
Tpm^Cdc8^-E129K	29.4	29.3, 38.1	24.5/26.2, 34.5	−331.1	−481.7, -403.6
Tpm^Cdc8^-E31K	32.2	35.9		−447.2	−571.3
Tpm^Cdc8^-A18TE31K	27.9	29.2, 43.2		−276.0	−256.8, -466.2

Unexpectedly when amino-terminally acetylated, both of the original temperature sensitive mutants, Tpm^Cdc8^.E129K (Cdc8.27) and the double mutation Tpm^Cdc8^.A18T/E31K (Cdc8.110), unfolded with two thermal transitions (Fig. 2D and E). Mass spectroscopy confirmed 100% of the purified mutant proteins were acetylated, confirming the double-transitions were not due to a mixed population of protein within the assay. The first transition had a midpoint similar to that of the unacetylated proteins (29.3 °C for E129K and 27.9 °C for the double mutation) and represented ∼60% of the transition in CD absorbance. The second transition was much more stable with T_m_s of 38.1 and 43.2 °C; 2.7 and 7.8 °C more stable than the wild type Tpm^Cdc8^ respectively.

**Fig. 3:**
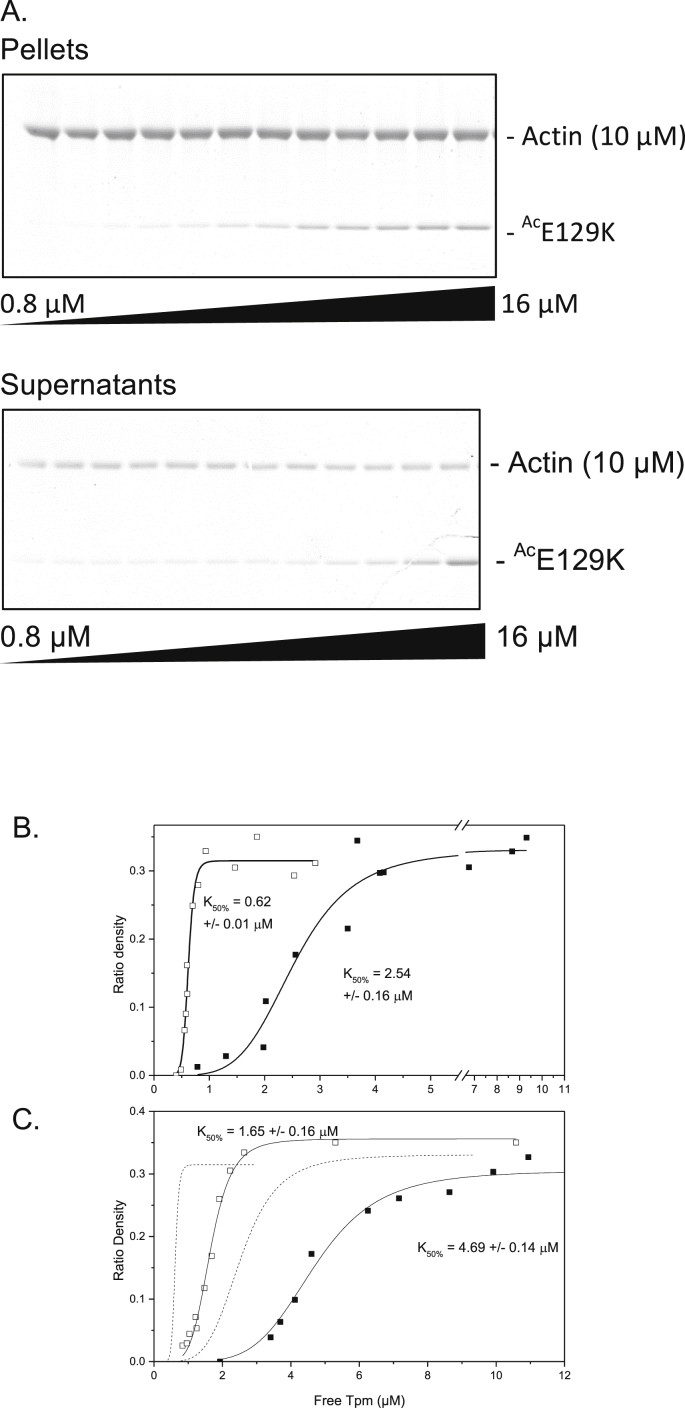
**Actin binding assays of Tpm**^**Cdc8**^. (A) Representive SDS/PAGE gels of pellet and supernatant fractions from the co-sedimentation assay of ^Ace^Tpm-E129K. Ratio of density was measured by densitometry of co-sedimentation SDS/PAGE gels. The free Tpm concentration plotted against the ratio of density of actin to Tpm from the SDS-PAGE gel for Nt-acetylated (empty shapes) and non-acetylated (filled shapes) (B) WT-Tpm^cdc8^ and (C) E129K-Tpm^cdc8^. Binding curves were generated by fitting the Hill equation to the data and the K_50%_ are the best fit values and standard error of the fit. Fits of wild type proteins (B) are shown in (C) as dotted lines for comparison.

The major function for Tpm within both muscle and non-muscle cells is to stabilise and regulate the function of actin filaments [3]. We therefore examined how a temperature sensitive mutation affected the ability of the Tpm to associate to actin. Actin-Tpm co-sedimentation experiments were conducted at 20 °C with WT and Tpm^Cdc8^.E129K. Tpm^Cdc8^.E129K was chosen because it not only has a low thermal stability, but also contains a single amino-acid substitution, predicted to lie on the surface of the subsequent coiled-coiled-coil dimer, and therefore may affect the interaction with actin. Fig. 3A shows representative coomassie stained SDS/PAGE gels from an Ac-Tpm^Cdc8.^E129K actin binding assay. The upper bands correspond to actin and remain approximately constant due to the fixed concentration of actin used and provide a loading control. The lower, faster migrating bands correspond to Ac-Tpm^Cdc8.^E129K, with the densities increasing from left to right, which corresponds to increase in Tpm concentration. This acetylated mutant Tpm has a high affinity for actin, as illustrated by the faint band observed in the pellet at 0.8 μM Tpm, and a much denser band is visible at 16 μM.

From these data, binding curves were generated, examples of which are shown in Fig. 3B-C. Consistent with previous studies published values unacetylated and Nt-acetylated forms of wild type Tpm^Cdc8^ have K_50%_ values of 2.54 ± 0.16 μM and 0.62 ± 0.01 μM respectively [27]. In contrast, Tpm^Cdc8^-E129K was observed to have a 2-3 fold lower affinity for actin compared to wild type, with corresponding K_50%_ values of 4.69 ± 0.09 μM and 1.65 ± 0.11 μM. Consistent with the phenotype and CD data, no evidence of binding to actin could be observed at 35 °C.

## 4 Discussion

As expected from the definition of the mutant Tpms as temperature sensitive the isolated protein showed loss of thermal stability in the expected range. As stated above, both amino-terminally acetylated and non-acetylated forms of Tpm are present in fission yeast and have distinct locations and roles in the cell [11], [12], [24], [28], [29], [30]. Considering the non-acetylated forms first since the effect of mutations is more straight forward to interpret. The wild type protein unfolds with a single thermal transition with a T_m_ of 34.1 °C. All three single point mutations resulted in a loss of stability; a 4.9 °C lower T_m_ for A18T and E129K and a smaller 1.9 °C shift for E31K (summarised in Table 1). The double mutant A18T/E31K had the largest reduction in T_m_ of 6.2° consistent with the effects of the two mutations being largely additive. These *in vitro* data are consistent with our phenotypic analysis of cell growth and AR dynamics in *cdc8*+, *cdc8.27* and *cdc8.110* cells (Fig. 1). While the *cdc8.27* cells displayed normal cell growth and cell division at temperatures up to 30 °C, *cdc8.110* cells displayed CAR formation and growth defects, even at the normal permissive temperature of 25 °C.

The almost >4.5 °C reduction in T_m_ from 34.1 C for non-acetylated A18T, E129K and the double mutant A18T/E31K would be sufficient to render most of the protein unfolded at 36 °C. If the T_m_ were similar in the cellular environment, much of the mutant protein would be unfolded at the permissive temperature for cell growth. For the single mutation E31K, which has not been shown to be a temperature sensitive mutation, the loss of stability is not as great and the cells are likely to remain viable at temperatures approaching 36 °C.

The CD studies described here were performed at 0.5 M KCl to prevent Tpm polymerizing end-to-end at the protein concentrations required for the CD measurements [27]. The end-to-end contact could provide additional stabilisation of the N & C-terminal regions in the Tpm polymer. However, little polymer is expected to form in the cell unless bound to actin. Reducing the KCl concentration to 0.1 M resulted in a 4.1 °C lower T_m_ for the non-acetylated wt Tpm^Cdc8^. This lower stability in 0.1 M KCl is consistent with hydrophobic interactions between the a & d positions in the heptad repeat being the primary driver for dimer formation. The loss of thermal stability was greater for the E129K mutant in 0.1 M KCl at between 4.9 °C. The environment of the cell is therefore likely to make the free Tpm even less stable than the values reported in Table 1.

Once acetylated the effects of the mutations are more complex. For wild type Tpm^Cdc8^ acetylation results in a 1.3 °C stabilisation of the T_m_ of the single unfolding transition of the protein. For A18T acetylation induces a similar 1.2 °C increase in T_m_ meaning the T_m_ remains 4.9 °C lower than the wild type. For E31K the stabilisation effect was larger at 3.7 °C, making the acetylated mutant marginally more stable than the wild type. This again suggests the single point mutation E31K on its own is unlikely to make the yeast cells temperature-sensitive.

For E129K and A18T/E31K acetylation results in a biphasic melting profile with ∼60% of the CD change occurring during the lower temperature transition. For E129K the lower transition occurs at a Tm of 29.3 °C, indistinguishable from that of the single transition of the non-acetylated form. For the double mutation the lower transition occurs at 29.2 °C, 1.3 °C higher than for the non-acetylated form and 4.2 °C lower then wild type acetylated.

The upper, smaller unfolding transition is significantly more stable with T_m_'s of 38.1 °C for E129K and 43.2 °C for the double mutant. These are significantly higher than the single unfolding transition T_m_ of the wild type protein (35.4 C). Since the major effect of N-terminal acetylation is a stabilisation of 40% of the Tpm it is likely this is the N-terminal 40% of the Tpm dimer. Since E129K and the double mutant disrupt the cell cycle at 36 °C this suggests this disruption occurs even though the more stable 40% of the protein will remain folded at this temperature. This could mean that the Tpm does not get rapidly cleared by the cell through proteolysis, but remains present in a semi-folded state allowing rapid refolding and reactivation if the temperature is lowered which permits refolding of the protein.

**Fig. 4:**
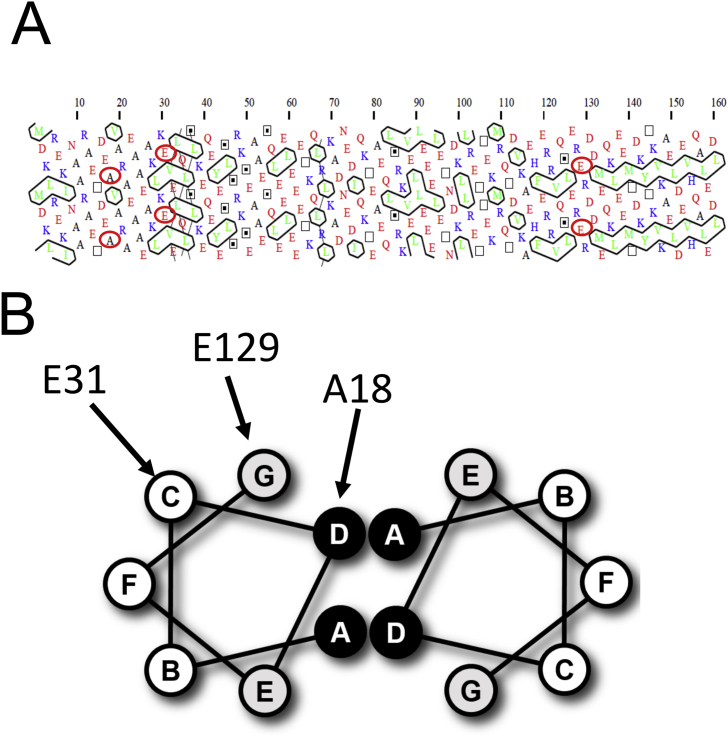
**Positions of the subsitituted residues.** (A) Hydrophobicity Cluster Analysis showing residues of an α-helix of Tpm^Cdc8^. The positions of mutations investigated in this study are shown circled in red. Red residues represent negatively charged amino acids, blue positively charged, and green hydrophobic amino acids. The black lines outlining hydrophobic patches indicate the hydrophobic core of the coiled coil. (B) Diagram illustrating a heptad repeat of 7 residues (A-G) between 2 α-helices. Residues in positions A and D are normally small compact hydrophobic residues involved in stabilising the coiled-coil interactions, and residues E and G are often charged and can form of a salt bridges between the two chains. (For interpretation of the references to colour in this figure legend, the reader is referred to the web version of this article.)

In the absence of a high-resolution Tpm structure it is difficult to give a precise interpretation of the destabilising effect of the mutations, but some features can be inferred from the position of the residue in the heptad repeat. Ala18 is in the core *d* position of the coiled-coil, normally occupied by small hydrophobic residues. An alanine in this position, with a single CH_3_- side chain, is thought to introduce some flexibility into the structure by reducing the density of side-chain packing compared to that in the classic leucine zipper coiled coil [5], [31]. In some vertebrate Tpms alanine appears in small clusters along the structure. The hydropathy plot of Tpm^Cdc8^ (Fig. 1B) shows A18 is part of an alanine cluster (A11-T15-A18.A22-A25) between two strong hydrophobic clusters (MLI, LVL: ringed in green in Fig. 4A). Replacing Ala18 with Thr introduces a larger side chain and some hydrophilic character into the core and also places two Thr residues close together. This is the probable cause of some destabilistation of the coiled-coil.

Glu31 is at a *c* position of the heptad and therefore thought to play little role in stabilising the coiled-coil structure. It does however sit next to K28 which will be positioned one turn of the α helix below E31 allowing a charge-charge stabilisation of the singe turn of the α helix. The E31K mutation would change this to a charge - charge repulsion causing some loss of stability but the effect of this single mutation is quite modest.

Glu129 is in the *g* position, where salt bridges between position *g* on one chain and *e* on the second chain (Fig. 4B) can give additional stability to the coil-coil. The equivalent *e* position is also a Glu making such a salt bridge impossible and the change to K129 might be expected to stabilise the coiled-coil. A closer look indicates that the local sequence is R128 E129 R130 with R130 in the *a* position of the heptad so this local structure is unlikely to conform to the standard coiled coil structure.

It is currently unclear why amino-terminal acetylation brings about a localised stabilisation of both the Cdc8.110 and Cdc8.27 proteins and not the wild type or single amino-terminal region mutants. The mutations in these proteins are located at opposing ends of the alpha-helical protein, and is therefore consistent with previous studies that have shown acetylation status and amino-acid modifications can have long distance effects upon the tropomyosin structure [27], [32], [33]. Indeed, each single amino-acid substitution is predicted to have no significant effect on the local structure of the alpha-helix (https://ppopen.informatik.tu-muenchen.de) or coiled-coil [34]. This signifies the mutations and post-translational modification are likely to bring about changes to the overall structure of the polypeptide or coiled-coil dimer. This is consistent with observations and models suggest that the conformation of the protein (i.e. dimerization, coiled-coil and polymer status) can bring about a significant (up to 3-fold) change in CD signal at 222 nm [35].

Nt-acetylation causes increased stability of all Tpms examined and for the two well defined temperature sensitive mutations separates the unfolding into two distinct regions. Since Tpm does not have distinct structural domains it would be incorrect to refer to these two unfolding transitions as domain unfolding but it is clear that there are two distinct regions that unfold with about 60% of the CD change occurring in the first transition. However, it is wise to be cautious about assuming that the CD signal has a strictly linear response to the amount of α-helix present.

## Appendix A Supplementary data

Fig. S1 **Morphology of temperature sensitive *Tpm***^***cdc8***^**alleles at 25ºC.** (A) GFP fluorescence and transmitted light images of mid log phase cultures of *cdc8*^+^, *cdc8.110*, or *cdc8.27* cells containing the *rlc1-gfp* allele grown at the permissive temperature of 25 °C for 48 h in YES. Asterisks highlight bent cells, with dotted lines denoting median axes of bent cells. Scale–5 μm. (B) Mean length and width of *cdc8*^+^, *cdc8.110*, or *cdc8.27* cells. Statistics were calculated from measurements of >300 cells of each strain. *- denotes t-test reveals differences at 99% confidence. Fig. S1

Fig. S2 **Electrospray mass spectra of amino-terminally acetylated Tpm**^**Cdc8**^**proteins.** Mass spectra of recombinant amino-terminally acetylated Tpm^Cdc8^ proteins, expressed and purified from *E. coli* confirm purity and homogeneity of samples. Predicted masses for each Nt-acetylated proteins are: Tpm^Cdc8+^-19,006.6; Tpm^Cdc8.A18T^–19,036.6; Tpm^Cdc8.E129K^–19,005.6, Tpm^Cdc8-E31K^–19,005.6; Tpm^Cdc8-A18TE31K^–19,035.7 Da. The non-acetylated protein should appear at a 42 Da lower mass – no such peak was observed. Fig. S2
