# Supplementary figures and images for: Temperature sensitive point mutations in fission yeast tropomyosin have long range effects on the stability and function of the actin-tropomyosin copolymer
Source: Biochem Biophys Res Commun. 2018 Nov 25;506(2):339–46. doi: 10.1016/j.bbrc.2017.10.109 (PMC6269162; doi:10.1016/j.bbrc.2017.10.109)

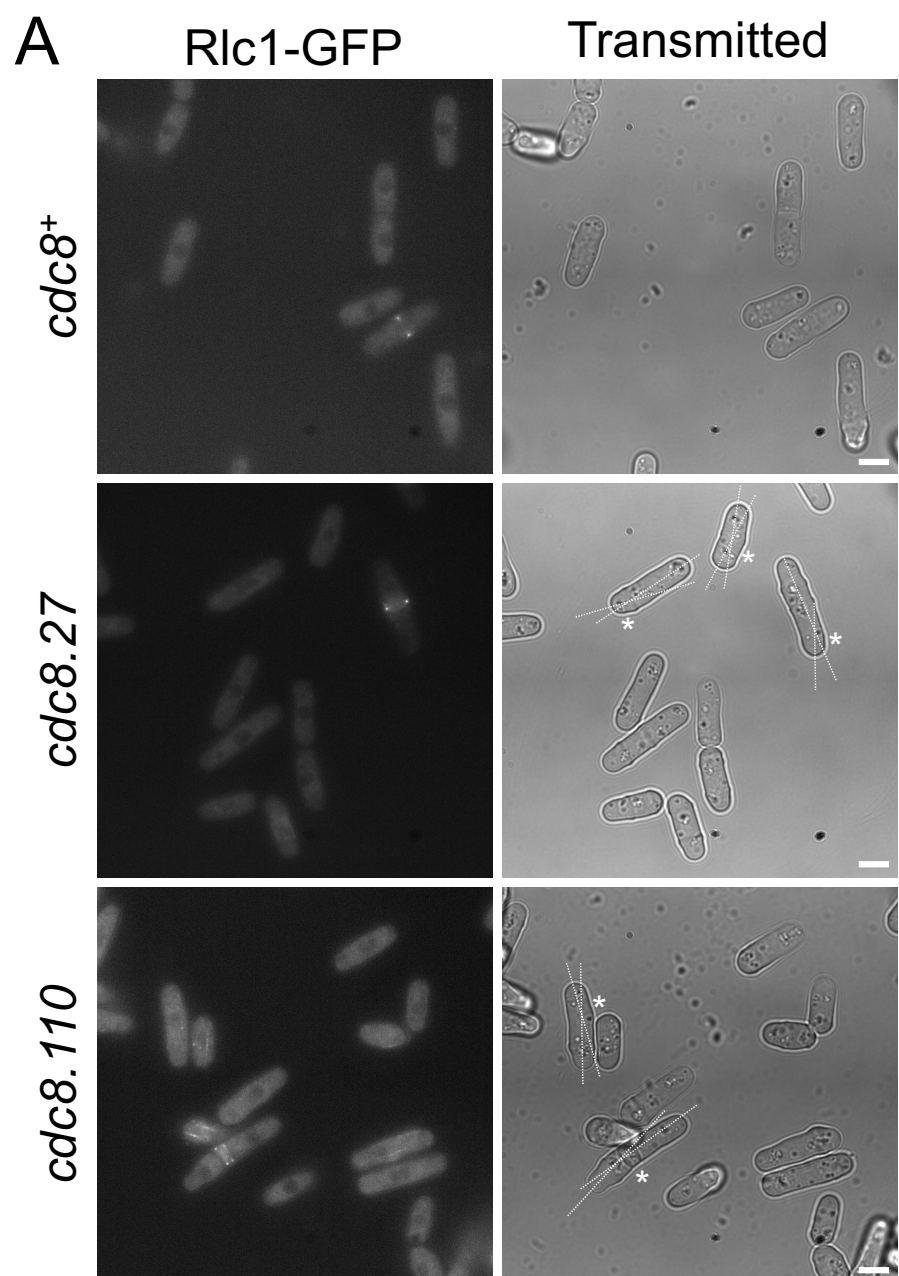

**B**

| Strain                  | Mean length (μm) | Relative width | n    |    |
|-------------------------|------------------|----------------|------|----|
| <i>cdc8<sup>+</sup></i> | 12.98 ± 2.37     | 1              | >600 | *] |
| <i>cdc8.27</i>          | 12.83 ± 2.65     | 1.01           | >300 |    |
| <i>cdc8.110</i>         | 11.67 ± 1.94     | 1.10           | >300 |    |

Figure S1

Supplement: Fig. S1 — Morphology of temperature sensitive Tpmcdc8alleles at 25ºC. (A) GFP fluorescence and transmitted light images of mid log phase cultures of cdc8+, cdc8.110, or cdc8.27 cells containing the rlc1-gfp allele grown at the permissive temperature of 25 °C for 48 h in YES. Asterisks highlight bent cells, with dotted lines denoting median axes of bent cells. Scale–5 μm. (B) Mean length and width of cdc8+, cdc8.110, or cdc8.27 cells. Statistics were calculated from measurements of >300 cells of each strain. *- denotes t-test reveals differences at 99% confidence. [file mmc2.pdf]

Tpm<sup>Cdc8</sup>

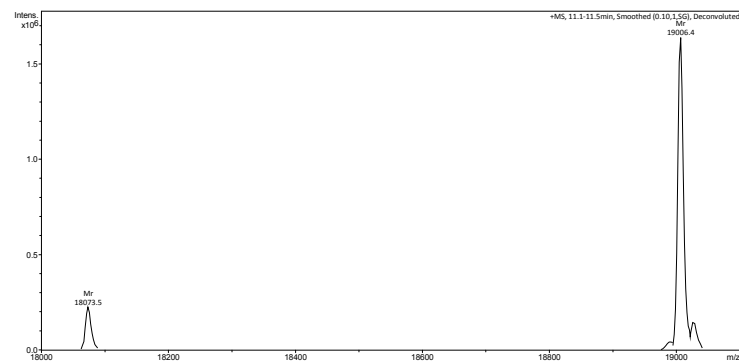

Tpm<sup>Cdc8-A18T</sup>

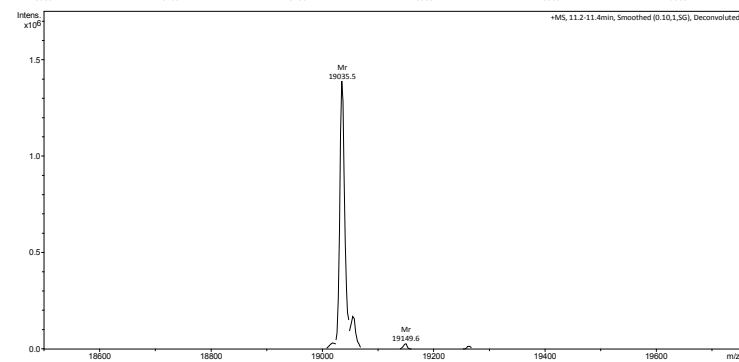

Tpm<sup>Cdc8-E129K</sup>

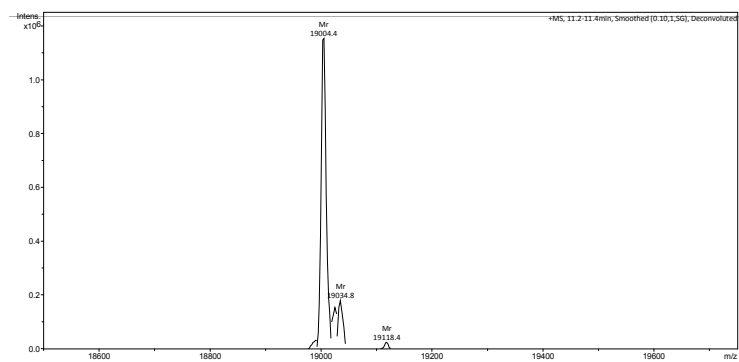

Tpm<sup>Cdc8-E31K</sup>

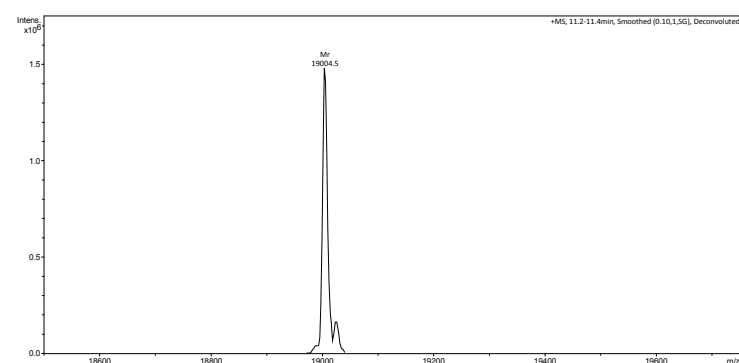

Tpm<sup>Cdc8-A18TE31K</sup>

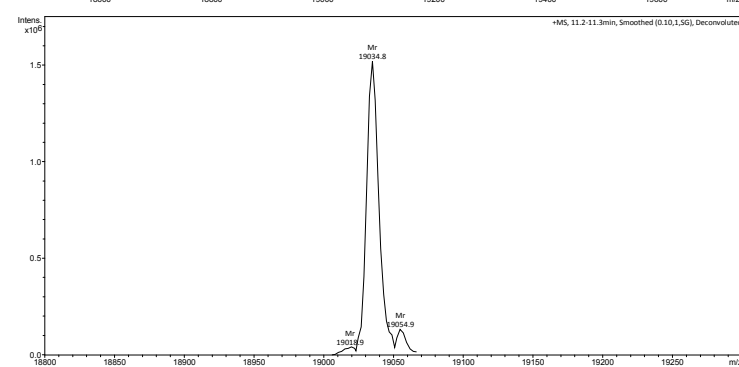

Figure S2

Supplement: Fig. S2 — Electrospray mass spectra of amino-terminally acetylated TpmCdc8proteins. Mass spectra of recombinant amino-terminally acetylated TpmCdc8 proteins, expressed and purified from E. coli confirm purity and homogeneity of samples. Predicted masses for each Nt-acetylated proteins are: TpmCdc8+-19,006.6; TpmCdc8.A18T–19,036.6; TpmCdc8.E129K–19,005.6, TpmCdc8-E31K–19,005.6; TpmCdc8-A18TE31K–19,035.7 Da. The non-acetylated protein should appear at a 42 Da lower mass – no such peak was observed. [file mmc3.pdf]
